# Social Media Use in Dermatology in Turkey: Challenges and Tips for Patient Health

**DOI:** 10.2196/51267

**Published:** 2024-03-28

**Authors:** Ayse Serap Karadag, Basak Kandi, Berna Sanlı, Hande Ulusal, Hasan Basusta, Seray Sener, Sinem Calıka

**Affiliations:** 1 Department of Dermatology Medical School of Istanbul Arel University Istanbul Turkey; 2 Private Clinic Ankara Turkey; 3 Department of Dermatology Medical School of Pamukkale University Denizli Turkey; 4 Department of Dermatology Medical School of Biruni University Istanbul Turkey; 5 Başusta Training and Consultancy Istanbul Turkey; 6 Pfizer Biopharmaceuticals Istanbul Turkey

**Keywords:** social media, dermatology, internet, health promotion, patient education, Instagram, YouTube, online social networking, social networking, Turkey, patient health, skin, skin disease, skincare, cosmetics, digital communication, misinformation

## Abstract

Social media has established its place in our daily lives, especially with the advent of the COVID-19 pandemic. It has become the leading source of information for dermatological literacy on various topics, ranging from skin diseases to everyday skincare and cosmetic purposes in the present digital era. Accumulated evidence indicates that accurate medical content constitutes only a tiny fraction of the exponentially growing dermatological information on digital platforms, highlighting an unmet patient need for access to evidence-based information on social media. However, there have been no recent local publications from Turkey analyzing and assessing the key elements in raising dermatological literacy and awareness in digital communication for patients. To the best of our knowledge, this study is the first collaborative work between health care professionals and a social media specialist in the medical literature. Furthermore, it represents the first author-initiated implementation science attempt focusing on the use of social media in addressing dermatological problems, with the primary end point of increasing health literacy and patient benefits. The multidisciplinary expert panel was formed by 4 dermatologists with academic credentials and significant influence in public health and among patients on digital platforms. A social media specialist, who serves as a guest lecturer on “How social media works” at Istanbul Technical University, Turkey, was invited to the panel as an expert on digital communication. The panel members had a kickoff meeting to establish the context for the discussion points. The context of the advisory board meeting was outlined under 5 headlines. Two weeks later, the panel members presented their social media account statistics, defined the main characteristics of dermatology patients on social media, and discussed their experiences with patients on digital platforms. These discussions were organized under the predefined headlines and in line with the current literature. We aimed to collect expert opinions on identifying the main characteristics of individuals interested in dermatological topics and to provide recommendations to help dermatologists increase evidence-based dermatological content on social media. Additionally, experts discussed paradigms for dermatological outreach and the role of dermatologists in reducing misleading information on digital platforms in Turkey. The main concluding remark of this study is that dermatologists should enhance their social media presence to increase evidence-based knowledge by applying the principles of patient-physician communication on digital platforms while maintaining a professional stance. To achieve this goal, dermatologists should share targeted scientific content after increasing their knowledge about the operational rules of digital channels. This includes correctly identifying the needs of those seeking information on social media and preparing a sustainable social media communication plan. This viewpoint reflects Turkish dermatologists’ experiences with individuals searching for dermatological information on local digital platforms; therefore, the applicability of recommendations may be limited and should be carefully considered.

## Introduction

Skin problems affect one-third of the general population worldwide, decreasing patients’ quality of life and adversely impacting their social lives [1]. Empowered by the widespread use of the internet, advances in mobile technology, digitalization of health care, and fundamental changes in interpersonal communication, patients with skin problems currently prefer to obtain prompt responses to health-related queries from digital platforms [2-5]. Therefore, seeking health information on the internet has become the predominant trend for patients in the digital age due to convenience, easy accessibility, anonymity, cost-effectiveness, and promotion of health equity [3,6]. Furthermore, patients seek convenient access to health tips at any time [1,7,8].

As one of the most prevalent health care resources, social media provides informative, “trendy,” and entertaining content independent of time and geographical distance [8-10]. Remarkably, social media networks, such as Facebook, Twitter, Instagram, YouTube, and TikTok, became more prevalent across all medical subspecialties during the COVID-19 pandemic [11]. Based on internet search data, the most frequently asked dermatological topics on social media networks include skincare, anti-aging, hair products, and acne vulgaris (AV) in Turkey and globally [7,12].

Relying on the information on social media and the limited social media presence of medical experts, patients are likely to make medical decisions based on posts created by individuals without medical or dermatological certifications [13]. These engaging posts may enhance the distribution of misleading information on social media, leading to harmful patient outcomes [9,14,15]. A digital survey study on hashtags from 9 dermatology-related Instagram posts with the highest number of followers reports that only 4%-5% of the hashtags were created by dermatologists registered with the American Board of Medical Specialties [14,16]. On the other hand, 93% of posts created by board-certified dermatologists were reported to have educational content for patients [16]. Recent reviews and studies have reported that most dermatology-related posts are prepared by individuals lacking formal training. Using engaging multimedia tools, these account owners substantially dominate most dermatological information on social networks [1,5,9,11,17-19]. According to a phone interview conducted in the United States, 1 in 3 US citizens use social media to search for information about health issues, and 46% of them are identified as “online diagnosers” [13]. Strikingly, 38% of these online diagnosers claimed they could handle their problems at home. The survey revealed that 82% of internet users aged 18-29 years sought health information on Google, Bing, Yahoo, or other search engines [13]. Another study revealed that 40% of internet users stopped taking their prescribed medications due to information on social media [20]. The national statistics showed that social media ranked first (80.9%) among reasons to use the internet in Turkey, while searching for health-related information ranked third (66.3%) [20]. In a nationwide study on social media use for AV, 70% of participants stated that dermatologists or dermatology associations should create posts about AV, and 51% agreed that only dermatologists should convey medical information on AV [7].

As an indispensable source for public health matters, social media may be an effective platform for dermatological outreach, facilitating access to evidence-based information and public education on dermatological issues in Turkey. To this end, it is necessary to identify the leading factors for dermatologists to maximize patient benefit through social media. However, there is a gap in local literature to guide dermatologists in establishing an effective social media presence. The expert panel aimed to collect expert opinions on identifying the main characteristics of individuals seeking health information related to dermatology and ways to increase patients’ knowledge and understanding of skin problems by creating evidence-based medical content on social media. Additionally, the panel aimed to address paradigms for dermatological outreach and define dermatologists’ roles in reducing misleading information on digital platforms.

## Methods

The goal was to form a multidisciplinary panel consisting of dermatologists and a social media specialist. A social media agency screened all popular social networks in Turkey, including Instagram, Twitter, and YouTube. The agency ranked the account owners who regularly posted dermatological content and had the highest number of followers in the past 6 months. Dermatologists without recorded professional credentials on social media accounts and those with a self-promotion or intervention promotion rate ≥20% per month were excluded from the candidate list.

The panel member candidates were selected according to the following criteria:

- Having professional credentials recorded on social media accounts
- Maintaining an active clinical practice
- Holding an academic title
- Having a high number of scientific publications
- Having a high number of followers on social media within the past 6 months
- Posting educational content regularly for the public (at least 3-4 posts per month)
- Achieving high engagement rates within the past 6 months.

Dermatologists who fulfilled these criteria were invited to participate in Pfizer’s project entitled “How to Use Social Media in Dermatology in Turkey.” A total of 4 dermatologists (2 from Istanbul, 1 from Ankara, and 1 from Denizli) accepted the invitation. To form a multidisciplinary expert panel, we sought a social media specialist who had relevant experience in creating communication strategies and managing crises on digital platforms and was experienced in conducting large-scale medical projects (not necessarily in dermatology) related to or on social media. After the final review of candidates, the social media specialist, who also held an academic position as a guest lecturer on “How social media works” at Istanbul Technical University, was invited to the expert panel.

Before the kickoff meeting, held on April 11, 2023, panel members were requested to review personal social media account statistics for characteristics of digital patients or health information seekers, identify the top 5 inquiry topics from the past 6 months, and read the national survey study about expectations of patients with AV from social media [7]. At the kickoff meeting, panel members agreed on 5 headings to be reviewed in the advisory board meeting (held on April 26, 2023). These headings were as follows: (1) general characteristics of Turkish health information seekers and preferred digital platforms in Turkey, (2) commonly inquired topics (eg, cosmetic vs medical dermatology and skincare vs treatment or interventional procedures), (3) algorithmic or digital communication parameters that may improve the dissemination of scientific knowledge on social media, (4) key elements of content creation for digital platforms or social media, (4) future perspectives of experts regarding social media impacts in dermatology. 

In the advisory board meeting, personal account statistics, experiences related to requests from information seekers, and problems caused by non–board-certified content creators (including physicians from other subspecialties) were discussed. The social media expert explained the cornerstones of digital communication and how to evaluate engagement quality on social media. In addition, dermatologists shared their experiences in effectively addressing the unmet needs of patients and disseminating clinically relevant information to health information seekers in dermatology across different digital media platforms on social media. Finally, the key recommendations resulting from these discussions were summarized and stated in this viewpoint.

### Characteristics, Behavioral Patterns, and the Unmet Needs of Turkish Dermatology Patients on Social Media

According to insights from participating dermatologists, approximately 60%-85% of dermatology patients were women aged 25-44 years with advanced digital skills who lived in megacities and used social media to obtain information on dermatological issues and choose a physician. Younger patients were more interested in cosmetic dermatology topics, whereas middle-aged patients often inquired about medical dermatology topics. In line with the digital data of 2023, the experts declared that they received nearly 90% of dermatology-related queries from Instagram [21].

In the present era, media and omnichannel communication have reshaped societal perceptions and self-perceptions of beauty standards [22,23]. As a result, young women consider self-image to be at the forefront of social acceptance. Under the pressing desire “to be within the societal beauty standards,” young adults are open to various cosmetic procedures and collect information mainly through digital platforms. Therefore, medical interactions on social media are slightly inclined toward cosmetic dermatology.

The leading causes of social media use among Turkish patients include seeking a diagnosis and learning about treatment options or procedures. Generally, a Google search is the first step in seeking dermatological information on the internet. However, most social media users are unfamiliar with the personalization algorithms of these platforms, which operate backstage to select and bring forth specific content according to previous search activities, labeling it as “recommended” or “suggested.” Moreover, depending on the topic, social media users often encounter massive amounts of information and have trouble determining whether the information is evidence based and relevant to their skin problems. With limited medical literacy in dermatology and digital competence, patients often perceive content shared on social media accounts with a “high number of followers” as “reliable.” However, most patients seeking information on the internet do not confirm whether the information is supported by scholarly sources or endorsed by dermatologists. Furthermore, it should be emphasized that individuals without professional training in dermatology cannot correctly categorize, evaluate, and discern accurate information by surfing websites, watching videos, and reading posts.

In cosmetic issues, especially, the procedure outcomes are presented with “before” and “after” photos, the majority of which are digitally edited. The medical content is prepared in a promising and appealing tone to draw the attention of nonmedical audiences. Therefore, dermatology patients should be vigilant and skeptical about medical content on social media lacking citations and making exaggerated promises about health outcomes. Inevitably, the absence of quality control and low levels of skepticism among social media users may lead them to websites broadcasting nonfactual information; this could delay access to effective treatment or result in patients experiencing complicated outcomes. Unlike in real life, patients have easy access to many websites via the internet, receive many recommendations, and use various skin products without consulting a dermatologist. Patients usually consult a dermatologist only when skin problems are not resolved with suggestions found on social media. Experts underlined the challenge of managing complex skin problems in daily clinical practice. Relying on overly promising posts and receiving dermatological treatment at later stages of the disease, patients have difficulty complying with more extended and comprehensive treatment plans. Additionally, it has been observed that the effectiveness of therapy is decreased in some cases due to delayed intervention.

According to recent national data, Turkey has a relatively young (median age 31.6 years), highly urbanized (77.2%) population, with equal gender distribution (women comprise 49.9% of the population) and an overall adult (age >15 years) literacy rate of 96.7% [21].

Some essential digital headlines of the Turkish population are as follows [21]:

- Internet access is available to 83.4% of the population.
- In total, 73.1% of the population are active social media users.
- Cell phone connection is available to 95.4% of the population.
- Approximately 85% of web traffic is from mobile phones.
- The population aged 16-64 years spends an average of 3 hours daily on social media networks.

**Figure 1:**
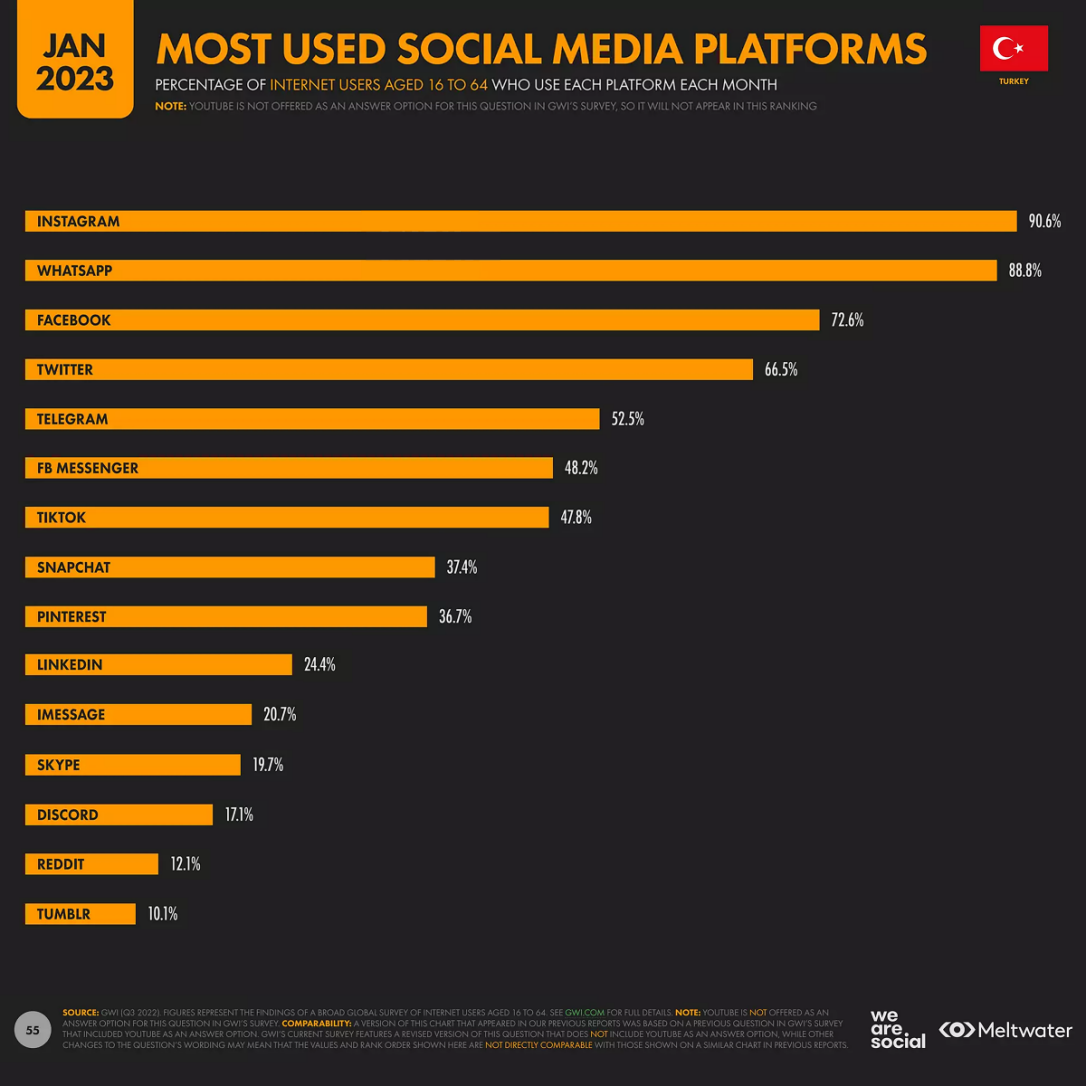
The most used social media platforms among the population aged 16-64 years in Turkey (reproduced from We Are Social & Meltwater et al [24], which is published under Fair Dealings as defined by Singapore Law according to copyright holder DataReportal [25]).

Since dermatology patients on social media are a subset of internet users in Turkey, it is imperative to delineate the essential patient characteristics. Instagram, WhatsApp, Twitter, TikTok, and Facebook are the most frequently used platforms, and with 90.6% of users, Instagram is the most popular social network in Turkey (Figure 1) [21].

**Figure 2:**
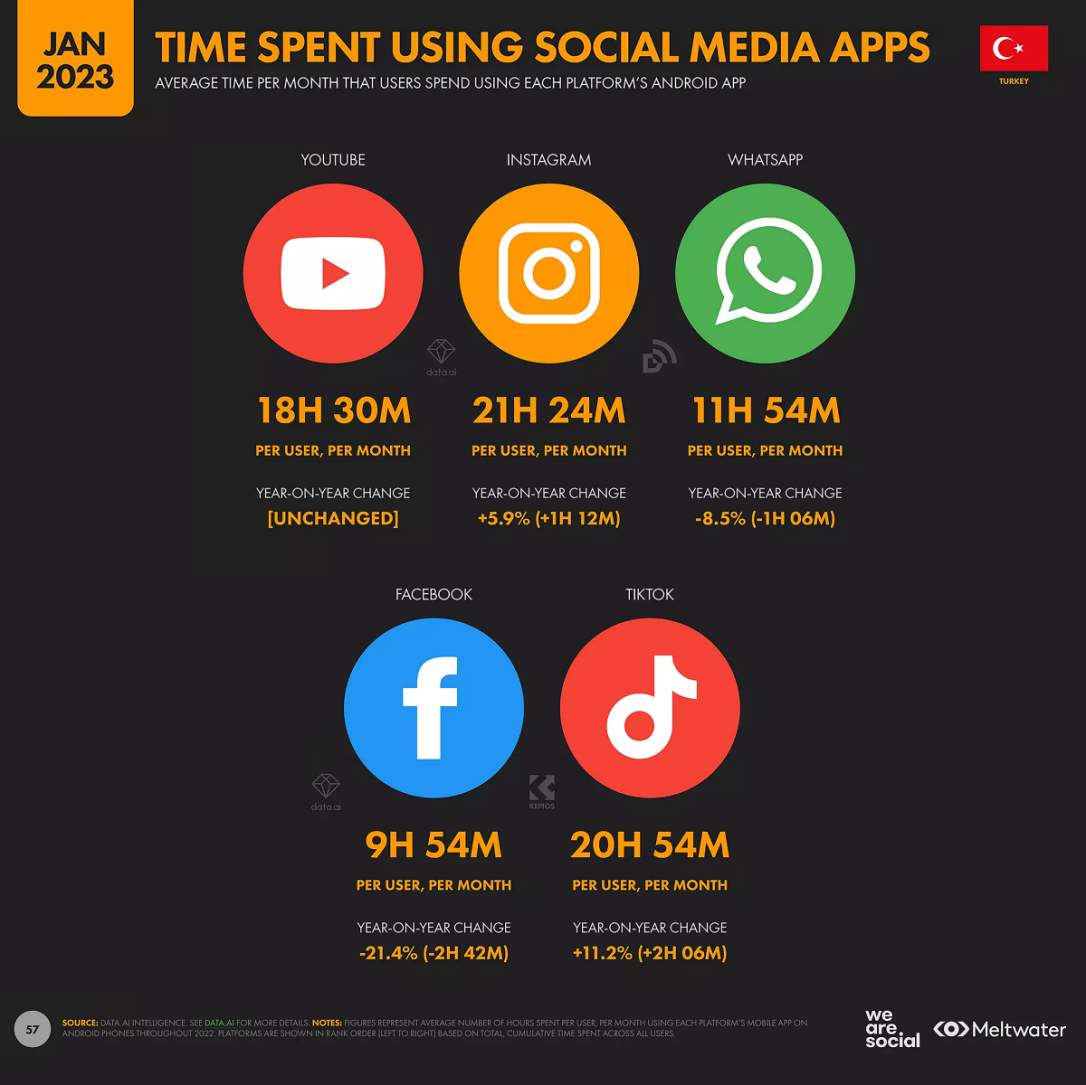
The average time spent per month on social media in Turkey (reproduced from We Are Social & Meltwater et al [24], which is published under Fair Dealings as defined by Singapore Law according to copyright holder DataReportal [25]).

The average time spent on Instagram per capita in Turkey was 20.2 hours per month in 2022, but it increased to 21 hours and 24 minutes in 2023 (Figure 2) [21].

**Figure 3:**
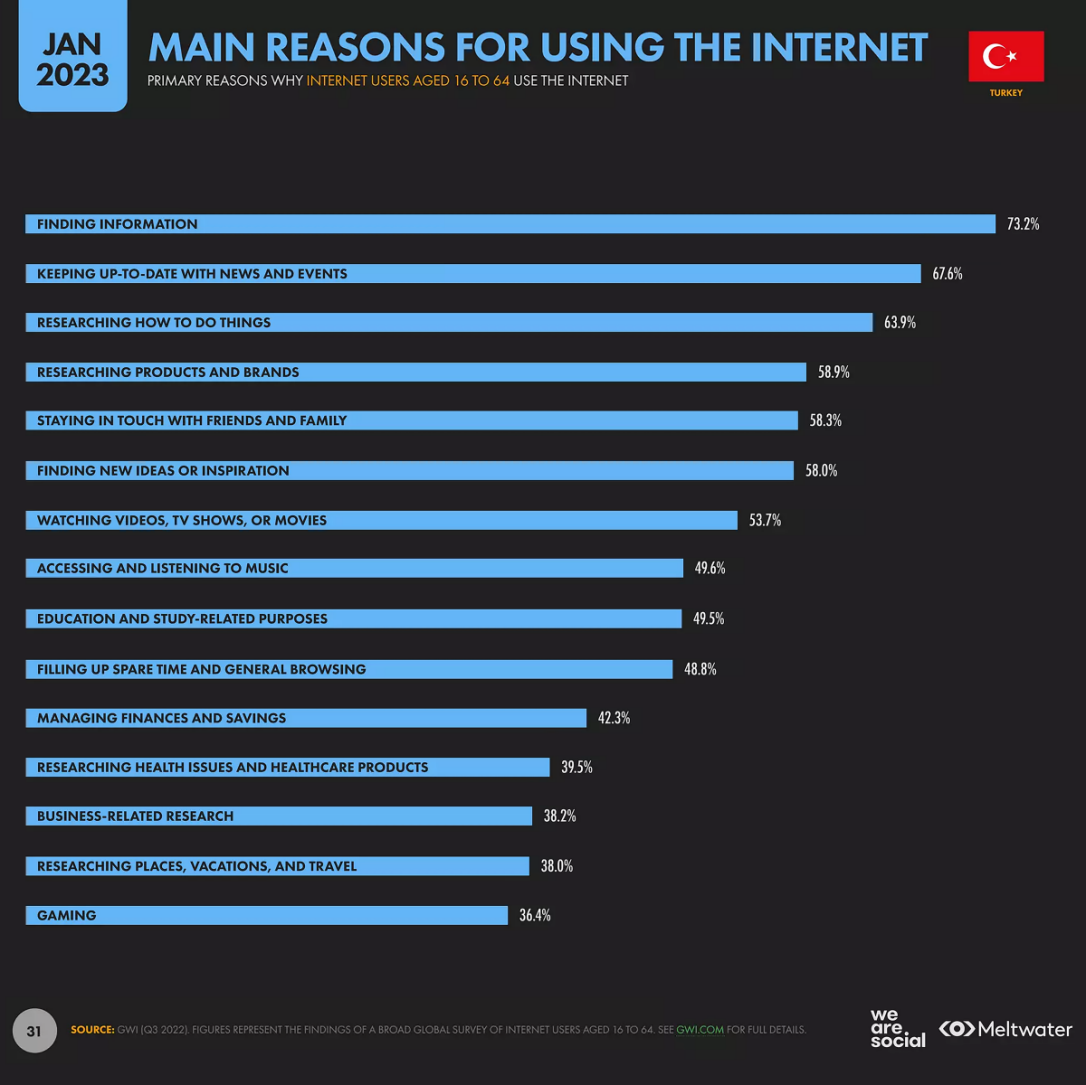
Reasons for internet use among the population aged 16-64 years in Turkey (reproduced from We Are Social & Meltwater et al [24], which is published under Fair Dealings as defined by Singapore Law according to copyright holder DataReportal [25]).

Of the population aged 16-64 years, 39.5% use the internet for “researching health issues and healthcare products” as one of the main reasons, and 17.7% follow beauty experts [21]. Figure 3 illustrates the reasons for internet use among the population aged 16-64 years in Turkey.

In a recent survey study on patients with seborrheic dermatitis, 81% of patients declared that they trusted the dermatologist’s decision on the treatment. However, 78.8% (104/132) also consulted social media for additional remedies for their diseases [26]. Among those who consulted social media, 54.8% started to use over-the-counter products, 35.6% implemented diet and lifestyle changes, and 7.7% started supplement use recommended on social media. The rest of the cohort (1.9%) started to use self-made products. Similar to our findings, 85.6% of those who consulted social media were female patients, and 78.8% were between the ages of 18 and 30 years. Instagram (63.6%) and YouTube (53%) were the most commonly used digital platforms for seeking health information [26].

### Non–Board-Certified Profiles Should Not Orchestrate Dermatological Outreach

Dermatological diseases are common, immediately noticeable, and generally nonfatal, yet they have similar symptoms. Although the skin is the largest organ in the human body, public awareness about skin health has increased only recently. It is a typical underestimation that most skin problems can be overcome without consulting a physician. Therefore, patients consult a dermatologist after unsuccessful self-treatment attempts, when the symptoms get worse and more problematic. Increasing patients’ health literacy is the cornerstone to improving patient understanding of cutaneous problems. In this respect, social media presents an excellent opportunity to disseminate scientific facts, as it readily engages large audiences without restrictions related to time and location.

Many studies have revealed that most individually prepared medical content is not based on scientific evidence or is occasionally entirely false [9,27]. For example, a recent cross-sectional analysis of a trendy topic, keratosis pilaris, on TikTok revealed that 52% of the content creators are nonphysicians, 16% are private companies, and 32% are physicians [28]. Interestingly, 16% of content-creating physicians were in medical branches other than dermatology (84% were dermatologists). In addition, a study characterizing the credentials of dermatology influencers on Instagram reported that board-certified dermatologists constitute only 4% of the Instagram accounts with popular dermatology content [29]. Furthermore, 71% of all influencers and 27% of health care influencers did not mention credentials on their accounts [29]. Finally, recent studies about effective communication on social media underlined that patients preferred dermatological content created by certified dermatologists and wished such content was more broadly available [7,9,19].

Dermatologists who want to participate in social media platforms should realize that in addition to patient education and heightened awareness, social media enables them to provide services they cannot offer in overcrowded outpatient settings. For example, sharing evidence-based information about routine skincare or aging will help patients on the internet differentiate scientific quality from temptation-provoking rhetoric in the content source. Furthermore, physicians can perform patient follow-ups more effectively, that is, they can communicate medication side effects and remind patients of medication use instructions. These efforts will soon lead to more medically literate patients in both the digital and real worlds.

Content creation, user engagement, and maintenance of digital patient communication require a time commitment, a budget to allocate a professional team, and follow-up on current advancements. Combating misleading dermatological information on social media is not a mission that the participation of a few dedicated dermatologists can accomplish. Instead, it demands the involvement of specialty associations, academic institutions, and reputable scientific journals to act in unison for the common objective. High-impact journals and leading institutions in dermatology have recently recognized the power of social networking worldwide, and therefore, activated social media accounts on different platforms [5,9,15,18,30,31]. There is an emerging need for Turkish patients to access verified information presented in lay summaries from scholarly sources. However, unfortunately, the social media presence of relevant institutions is far behind what is desired in Turkey.

The level of social media presence varies mainly according to age and the perception of dermatologists toward social media. Being exceptionally acquainted with digitalization early in life, younger dermatologists are more competent with the algorithms of digital platforms and eager to build a successful career, including maintaining a strong social media presence. On the other hand, being active on social networks may be controversial for more experienced dermatologists and academicians. There may be various underlying reasons, such as timidity in communication in the digital world, incompetence with digitalization, and discomfort with or prejudice against social networks due to everyday use. Furthermore, the social media presence of dermatologists may depend on the institution in which they work. In Turkey, private hospitals use social media extensively to broadcast information about diseases, procedures performed, and technical expertise.

### What Matters in Social Media Presence? Roles of Dermatologists

Dermatologists have a leading role in high-quality health information available on the internet. It should be remembered that whether in the digital or real world, a physician is a respected, trusted source of medical information for patients and a role model for the next generation of physicians as well as colleagues. Considering social platforms as meeting points for academia and the general population, dermatologists need to fulfill their scientific roles in disseminating reliable information. In contrast to this, most social media account owners who have a substantial impact on patients are not health care professionals. Some patients have noted that they follow popular profiles for “fun” but do not rely on them to solve critical health issues. Moreover, a survey about patients’ self-reported trust in physicians based on their behaviors on digital platforms has shown that social media users prefer professionalism in essential matters such as health [32]. Therefore, it should be noted that it is difficult to earn trust and to reestablish it when it is lost. A physician-patient relationship built on medical facts will result in guidance and permanent engagement with most patients and health information seekers on social networks, eventually counteracting misleading content by disseminating accurate information. Therefore, dermatologists should preserve their professional stances on social media despite the complexities of digital platforms.

Since dermatology is one of the hot topics on social networks, most content creators in this field need help in their leadership role in the cosmetics market, which can readily promote unrealistic promises and propagate harmful trends. As a result, globally and locally, academic institutions, medical associations, and legal authorities have recently passed professionalism policies to regulate digital medical communication and preserve patient privacy and confidentiality (Textbox 1) [33-36]. It should be noted that policies and codes of ethics for social media use should be applied to every account owner who broadcasts health-related content.

**The American Medical Association’s recommendations for social media use.**

**Physicians should consider the following:**

- Be cognizant of patient confidentiality and refrain from posting any identifiable patient information online.
- Use privacy settings to safeguard personal information on social networking sites.
- Maintain appropriate boundaries of the patient-physician relationship following professional ethics guidance.
- Consider separating professional and personal content online.
- Recognize that content shared online may have negative impacts on their reputation.

### Essentialities in Social Media Contact and Content Creation

The underlying essential elements of effective communication on social media include a well-structured strategy, content creation, and engagement metrics. When creating content, the device(s) on which the content will be shared should also be considered; for instance, if most website traffic comes from smartphones, posts should be mobile-friendly.

**Figure 4:**
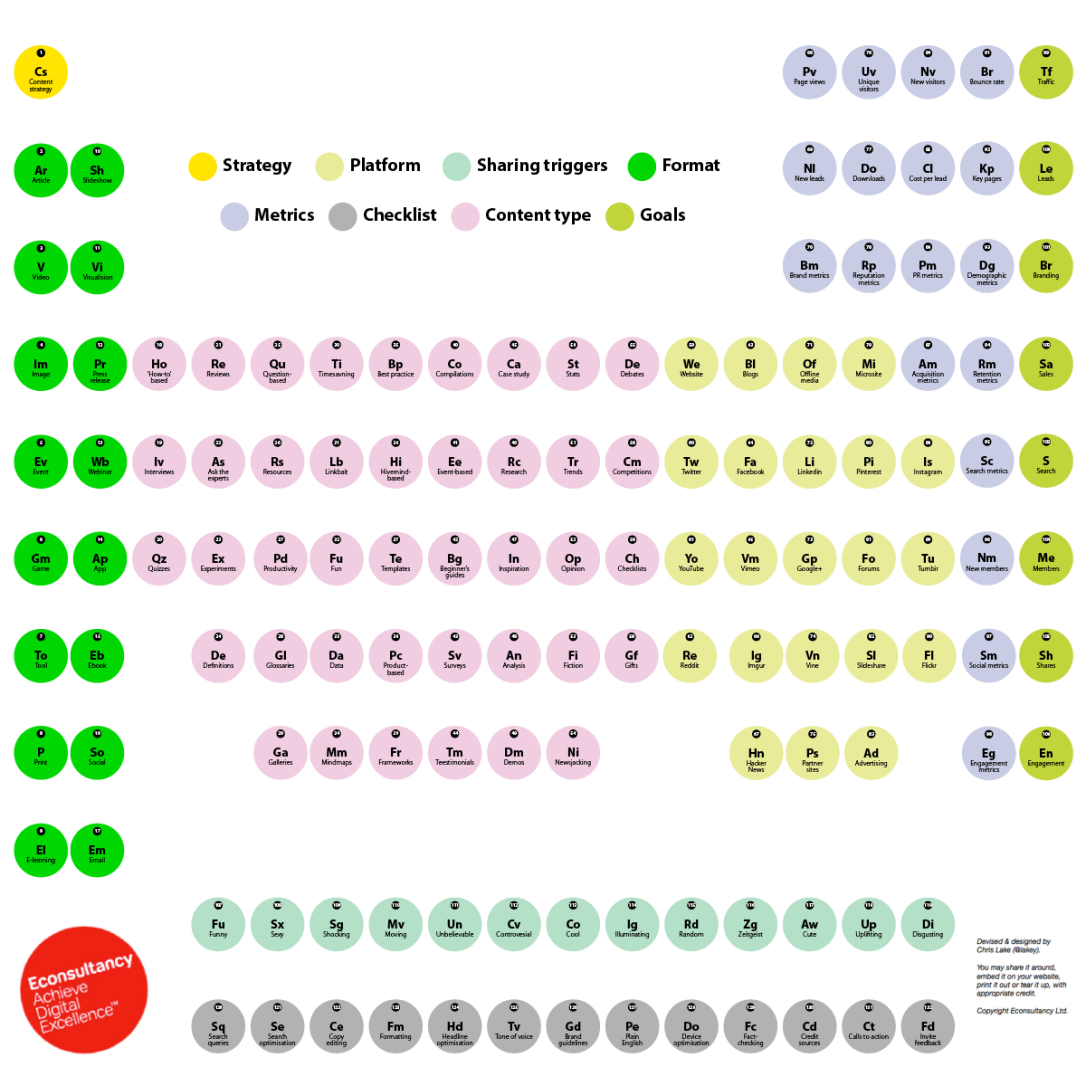
Key elements of content creation.

Searching for accurate information is the primary driver of social media use for most dermatology patients. Therefore, broadcasting on social media is crucial to increasing factual dermatological knowledge on digital networks [37,38]. The panelists believe that dermatologists with predefined strategies before broadcasting on social media will have successful engagement rates. The strategy should be individualized for the target population with the help of search engine optimization workups. Some steps, outlined in the following sections, should be carefully defined before posting content on digital platforms, including the platform selection, format, and multimedia tools to use. The panelists believe that the most straightforward, yet practical steps of content creation and sharing are summarized in Figure 4 [37].

Broadcasting informative content involves the selection of the social media platform and content topics as well as the frequency of broadcasting. For a dermatologist, content creation is a time-consuming process, as it requires literature review, rewriting for the target audience, preparation of visuals, and editing according to the format of the digital platform.

Most new social media account owners prefer to stay socially active by copying content they like from other accounts. However, such an approach can facilitate the dissemination of misinformation from the original site, causing the account holder to lose patients’ trust and increasing the cited site’s rankings by transmitting linked tools. In fact, patients understand and prefer authentically created content in lay language. With transparency, professionalism, and clear boundaries, dermatologists should refrain from overpromising results to attract attention; instead, they should be open to receiving negative reviews and accept them as part of digital communication.

Not all followers who use social media are patients or advice seekers. One out of 10 followers is reported to write intentionally provocative and offensive messages on social media and is labeled as a troll [39]. Keeping up with the rules of physician-patient communication upheld in face-to-face settings on digital platforms may be a practical way to sustain professionalism on these platforms [39].

#### Content Creation

Content creation involves selecting the topic and the platforms (Figure 4) [37]. Digital tools provide keywords (hashtags) with high search volumes on the internet. Therefore, informative content that guides patients should be included in them so that the content can be more readily shown to other relevant searchers. They can be provided by periodically used search engine optimization workups.

Cross-posting is an effective measure to reach a broader audience on social media. Sharing the same content on different platforms with specific interfaces enables influencing more patients, staying up-to-date, and saving time. It is observed that Turkish patients on social media prefer short videos and a combination of videos and images, namely carousel posts, on dermatological topics. Shooting less-than-1-minute videos is generally recommended to maintain the audience’s attention. For written content, catchy titles, including relevant keywords (hashtags), attract patients’ attention. Informative content should be in lay language with simplified explanations of medical terms to support patient engagement. The posting frequency depends on how much time the dermatologist can spend on the networks.

#### Increasing Patient Engagement

Social media metrics serve as data points to evaluate the quality of digital communication. Engagement metrics guide content creators in identifying posts that resonate better with followers by providing data on session durations, page views, conversion rates, and followers’ feedback. These metrics can reveal areas for improvement in previous posts, including deficiencies in content writing or images, to enhance future ones. With the help of engagement metrics, patient behavior on digital platforms can be better determined to build effective communication.

#### What Lies in the Future

Experts note that emerging technologies and tools, especially artificial intelligence and applications for better photography in teledermatology, have significantly increased diagnostic accuracy. Considering the present technological advances, one may expect the computer systems to perform tasks that require human intelligence, such as skin lesion classification, improving the diagnosis and management of psoriasis, assessing ulcer specifications, and early evaluation of skin cancer via artificial intelligence–based machine learning and convolutional neural networks [40,41]. For example, the recently launched ChatGPT has already become very popular in writing patient care discharge summaries and promoting healthy lifestyle practices. During the pandemic, teledermatology increased worldwide to ensure patients and health care providers had access to dermatologists [42-44]. Moreover, teledermatology enabled patients from remote areas to obtain access while reducing wait times for dermatology referrals [45-47]. Recent advances have improved the quality of smartphone photos, and dermoscopy through smartphone microscope apps using convolutional neural networks is increasingly applied in teledermatology consultations [48].

It should be foreseen that advances in the field of technology will continue at a rapid pace. Nevertheless, increasing digital possibilities will allow people with dermatological problems to access medical information from more channels. Therefore, policies and codes of ethics for health care topics should be implemented in all aspects of the digital world as soon as possible. Dermatologists, academic institutions, and specialty associations should take their place in disseminating dermatological knowledge on social media networks.

### Conclusions

The experiences of Turkish dermatologists with a strong social media presence and inquiries from patients on informative digital channels indicate that medical advice-seeking individuals need help accessing scientific information. Therefore, dermatologists who master this field should become critical players in the dissemination of accurate knowledge and in raising public awareness in digital settings. Recently accumulated local data on the impact of social media on dermatology-related professions have pointed out that patients or health information seekers are misguided by unconfirmed and unmonitored digital content. Given that the digital world will soon be much more indispensable in dermatology and patient-physician relationships, combating misleading information created by nonqualified account owners should be a shared responsibility of dermatologists, academic institutions, and board associations. The authors of this paper state that increasing the digital operational competence of dermatologists, while complying with the ethical rules of the medical profession, is the cornerstone for disseminating evidence-based information and patient awareness in the field of dermatology on digital platforms. For the highest benefits of health information seekers, social media presence rates of institutions and dermatologists should be increased collaboratively.

### Key Recommendations

Some key recommendations drawn from this study are as follows:

- Dermatologists should be authentically present on social media within their medical profession.
- The communication on social media should be aimed at establishing a reputable, enlightening, and reliable patient-physician relationship on digital channels.
- Patient communication should be direct, natural, sincere, and convincing, rather than artificial.
- Patients’ confidential information must not be shared in any way, and patient privacy must be meticulously protected.
- Raising patient awareness and combating misleading information should be the primary goal of the digital presence of dermatologists.
- Content should be original and created with proven data. Content should be brief for easy understanding.
- Posts should support patient feedback, and the access rate should be measured with metrics.
- Content interaction should be periodically evaluated.
- Cross-posting in different social media channels provides uninterrupted patient communication.
- Academic institutions and associations need to be more involved in digital platforms.

## Abbreviations

AV: acne vulgaris
